# Processing biological literature with customizable Web services supporting interoperable formats

**DOI:** 10.1093/database/bau064

**Published:** 2014-07-08

**Authors:** Rafal Rak, Riza Theresa Batista-Navarro, Jacob Carter, Andrew Rowley, Sophia Ananiadou

**Affiliations:** ^1^National Centre for Text Mining, School of Computer Science, University of Manchester, M1 7DN, UK and ^2^Department of Computer Science, University of the Philippines Diliman, Philippines 1101

## Abstract

Web services have become a popular means of interconnecting solutions for processing a body of scientific literature. This has fuelled research on high-level data exchange formats suitable for a given domain and ensuring the interoperability of Web services. In this article, we focus on the biological domain and consider four interoperability formats, BioC, BioNLP, XMI and RDF, that represent domain-specific and generic representations and include well-established as well as emerging specifications. We use the formats in the context of customizable Web services created in our Web-based, text-mining workbench Argo that features an ever-growing library of elementary analytics and capabilities to build and deploy Web services straight from a convenient graphical user interface. We demonstrate a 2-fold customization of Web services: by building task-specific processing pipelines from a repository of available analytics, and by configuring services to accept and produce a combination of input and output data interchange formats. We provide qualitative evaluation of the formats as well as quantitative evaluation of automatic analytics. The latter was carried out as part of our participation in the fourth edition of the BioCreative challenge. Our analytics built into Web services for recognizing biochemical concepts in BioC collections achieved the highest combined scores out of 10 participating teams.

**Database URL:** http://argo.nactem.ac.uk.

## Introduction

A number of frameworks have been developed and adopted to alleviate issues of interoperability between various biomedical text-mining solutions. The General Architecture for Text Engineering (GATE) (1) offers a family of open-source text processing tools, including GATE Developer, Embedded, which provides access to a rich library of interchangeable components that can be integrated with user-defined applications. The GATE framework has been recently applied to gene-associated studies, drug-related searching and medical record analysis (2). Another framework, the Unstructured Information Management Architecture (UIMA) (3), promotes the interoperability of data processing components by defining common data structures and interfaces. With the framework gaining popularity over the past decade, several UIMA-compliant repositories have been developed, including those of U-Compare (4), META-SHARE (5), DKPro Core (6) and JCoRe (7), and others that are focussed on biomedical solutions, e.g. the clinical Text Analysis and Knowledge Extraction System (cTAKES) library (8) and the BioNLP-UIMA component repository (9).

Supporting these frameworks are workflow construction platforms that allow users to build customizable natural language processing (NLP) solutions based on workflows comprising interchangeable components. GATE users, for instance, use the integrated development environment GATE Developer in setting up pipelines with task-specific plugins. Similarly, the stand-alone platform U-Compare provides its users with access to a library of UIMA components, from which NLP solutions, realized as workflows, can be built. The wide applicability of such solutions, however, is often hindered by the workflows’ software dependencies on the source platforms. While most of them provide import and export mechanisms to foster the sharing of workflows, they are usually interchangeable only within the same platform. Furthermore, their integration with other systems becomes a non-trivial task, often requiring additional programming effort.

For these reasons, solutions deployed as Web services have become more widely used and accepted, owing to their public availability and conformance to standards, e.g. Representational State Transfer (REST) architecture. They are easily accessible to users from libraries such as the BioCatalogue (10), a registry of Web services for the life sciences, and Whatizit (11), a suite of biomedical concept recognition services. The drawback, however, of implementing solutions as Web services, is the requirement for programming knowledge and effort.

With these considerations, it becomes desirable to develop solutions while combining the strengths of workflows and Web services. On the one hand, building tools as workflows based on interchangeable processing components not only eliminates the need for programming effort, but also allows for customization in terms of underlying analytics and supported formats. Deploying them as Web services, on the other hand, promotes cross-platform interoperability and far-reaching applicability. These desiderata motivated the development of Web service deployment extensions to scientific workflow construction platforms such as Taverna (12), Kepler (13) and Triana (14), as well as U-Compare (15).

In parallel with the advancement of various frameworks, repositories and platforms are efforts aimed at establishing data interchange formats for encoding information. Pioneer work towards this end include the first stand-off annotation format from the TIPSTER Common Architecture (16) and the abstract XML representation from the Architecture and Tools for Linguistic Analysis Systems, which incorporated annotation graphs (17). Later on, the ISO/TC37/SC4 standard-compliant Graph Annotation Framework format was also developed (18).

In encoding documents and annotations from the domain of biomedicine, more widely used and adopted formats, namely those of the XML Metadata Interchange (XMI) and Resource Description Framework (RDF), have been used. The Colorado Richly Annotated Full Text corpus (19), for instance, was distributed in both formats, while the CALBC silver standard corpus was encoded in the latter (20).

Biology-specific formats have also been introduced. The BioNLP Shared Task series (http://2013.bionlp-st.org, http://2011.bionlp-st.org, http://www.nactem.ac.uk/tsujii/GENIA/SharedTask), which fosters community efforts in developing solutions for fine-grained, biology-related information extraction, proposed their own format for sharing data with participating solution providers (hereafter referred to as BioNLP format).

The BioC format was actively promoted by two tracks of the fourth edition of the BioCreative workshop (21, 22) in its aim to advance the reusability of resources as well as the interoperability of tools and Web services.

In this article, we describe customizable Web services that support interoperable formats and are capable of extracting various biologically relevant concepts and interactions in a given data source. The mentioned customization of Web services is two-fold: (i) users are free to design their own processing pipelines from a repository of analytics, and (ii) the pipelines may be set up to accept and produce a combination of input and output data interchange formats. We focus on two domain-specific formats, namely, BioC and BioNLP, as well as generic formats, XMI and RDF. The entire process is realized in the text mining workbench Argo. A selection of the features of Argo relevant for this work is listed and compared against aforementioned solutions in Table 1.

**Table 1.** Comparison of selected functionalities of Argo and other related platforms

Feature	Argo	GATE Developer	U-Compare	Taverna	Kepler	Triana
Based on a standard interoperability framework	+	−	+	−	−	−
Web-based	+	−	−	−	−	−
GUI-based workflow construction	+	+	+	+	+	+
In-built library of analytics	+	+	+	−	+	+
Focussed on text mining	+	+	+	−	−	−
Strong support for biomedical applications	+	+	+	+	−	−
Support for data curation	+	+	−	−	−	−
Workflow sharing	+	+	+	+	+	+
Web service deployment	+	−	+	+	+	+
Customizable I/O formats for Web services	+		−	−	−	−

In the remainder of this article, we present a discussion of how Web services are customized and deployed from workflows created using Argo, followed by an overview of the various supported file formats. To illustrate, we provide two examples of Web service-enabled workflows performing automated processing of diversely encoded biological documents. We next describe the corpora that we have made available to the community as supporting resources. We also provide a thorough description and report on the results of our participation in the Comparative Toxicogenomics Database (CTD) track of BioCreative IV, which served as a systematic evaluation of our Web-service methods. We conclude by summarizing our contributions and discussing the limitations of proposed formats in terms of interoperability.

## Customizable Web services

The two types of customization of Web services, i.e. designing users’ own processing pipelines and choosing a combination of input and output formats, are realized in our text-mining workbench Argo. The workbench is a Web-based platform that allows users to collaboratively design and evaluate text-mining workflows (23). The workflows are created in a graphical user interface resembling block diagramming. Each workflow is an arrangement of a selection of available elementary processing components or analytics. The most common arrangement is a pipeline in which processing of input data is carried out in a series of subsequent steps, where each step is an elementary component. The available components range from data deserializers (opening a workflow and ingesting input data) and serializers (closing a workflow and producing output data) to NLP components to semantic analytics, such as named entity recognizers and entity-interaction extractors. Argo features several data deserialization and serialization components, or readers and writers, that are Web service-enabled, i.e. the presence of these kinds of components in a workflow facilitates its deployment as a Web service.

A single workflow, therefore, ultimately governs the 2-fold Web service customization process. The selection of Web service-enabled readers and writers imposes the input and output formats of the service, whereas the other components enclosed by the reader and writer define a processing task.

The interoperability of processing components in a workflow is ensured by UIMA by means of common interfaces and data structures. Components exchange common annotation structures (CASes) whose semantics is governed by flexible, well-defined and developer-expandable type systems (annotation schemata).

Each execution/processing of a workflow deployed as a Web service is assigned a unique URL that becomes the service’s access point. Users can track the progress of processing as well as gain access to their generated URLs via Argo’s interface.

## Supported formats

Argo currently includes several Web service-enabled readers and writers that support generic formats, XMI and UIMA Resource Description Framework (UIMA RDF), as well as formats used in biological literature processing, BioC and BioNLP.

XMI, an industry standard for exchanging metadata information, is an XML-encoded, stand-off format in a sense that annotations about data are not located in-line (i.e. within the data). XMI is popular as a data-exchange format in UIMA applications because of the fact that the open-source, Apache-licensed implementation of this framework features utility tools for serializing and deserializing data into and from this format (http://uima.apache.org).

UIMA RDF, a result of our earlier work (24), is essentially an RDF representation of UIMA’s CASes. It uses RDF Schema (http://www.w3.org/TR/rdf-schema) as the underlying vocabulary that is suitable to fully express UIMA structures, such as a hierarchy of types, their instances and relationships between them; it also forms a base for semantic languages such as OWL.

BioC (25), an emerging XML-encoded format, represents a collection of documents by interweaving in-line annotations with stand-off annotations. In-line annotations include the segmentation of document’s text into passages and optionally sentences. Stand-off annotations can be embedded into these elements, allowing for the inclusion of information such as text-bound locations and *n*-ary relations between annotations. Virtually all allowed XML elements may include structures for defining a list of key-value pairs.

In the BioNLP format, both source text and annotations are encoded in plain text and are kept in two separate files. Annotations include named entities and biological processes or events, i.e. named relationships between an event trigger word and other named entities and/or events.

To complete the possible combinations of input and output formats for Web services, Argo also features a reader that simply accepts data in plain text.

## Workflows for processing biological literature

Below, we describe two examples of workflows prepared in Argo that can be deployed as Web services. The workflows process biological literature and extract biologically relevant concepts and interactions. The first workflow features homogenous input and output formats, whereas for the second workflow the input and output formats are different.

### Identification of metabolic process concepts

**Figure 1.**
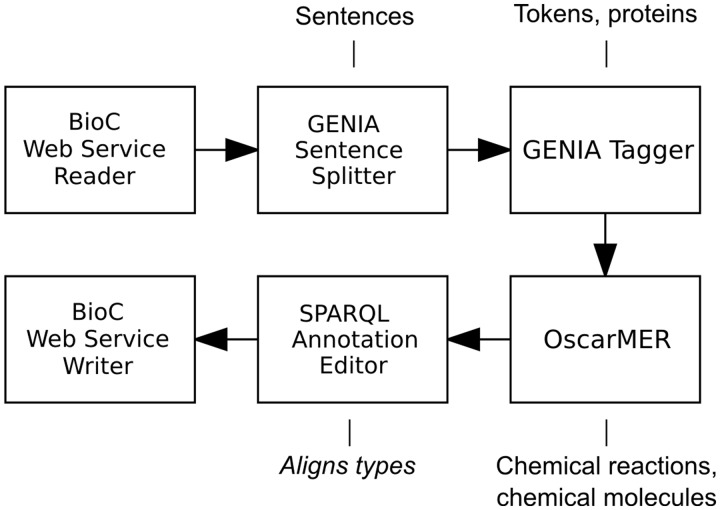
A Web service-enabled workflow built in Argo for identification of metabolic process concepts. The workflow features BioC as the Web service’s input and output format. The callouts show component-specific output annotation types that are relevant for this workflow.

The identification of biologically relevant concepts pertaining to metabolic processes was a biocuration task that we defined as part of our participation in the interactive track of the BioCreative IV challenge (26). The task involved the annotation of gene or gene products and chemical compounds, as well as action words (verbs, verb nominalizations or adjectives) signifying an occurrence of a metabolic process involving the two concept types in a selection of PubMed abstracts. The presented workflow, also illustrated in Figure 1, is a simplified version of that used in the BioCreative challenge.

The workflow is meant to update an input BioC collection that may include any biologically relevant text and annotations. Input and output are handled by the BioC Web Service Reader and BioC Web Service Writer components. The reader converts the BioC-compliant XML input data by separating the source text from the BioC annotations. Each document is then segmented into sentences and tokens by the GENIA Sentence Splitter and GENIA Tagger, respectively. Automatic recognition of metabolic process concepts is performed by the built-in GGP recognizer of the GENIA Tagger and OscarMER that recognizes chemical compounds and action terms. Both components use machine learning models. The SPARQL annotation editor component (24) is used to align types between the BioC type system and the type systems supported by the other components. The writer performs the opposite conversion to that of the reader.

### Biological event extraction

The extraction of events in biological literature is the subject of the BioNLP Shared Task series. An event is defined as a structure consisting of a typed trigger word or phrase signifying a biological process (e.g. ‘activation’, ‘inhibits’) and participating entities labelled with semantic roles (e.g. ‘theme’, ‘cause’). Events may also be enriched with attributes (e.g. negation and speculation) that modify their interpretation. Furthermore, event annotations may include information pertaining to the equivalence or coreference between expressions such as abbreviations and their corresponding expanded forms. The task, as it is defined in the shared task series, is to process documents that already contain the annotations of biological concepts (named entities) and produce annotations pertaining to event triggers, participants and modifications.

**Figure 2.**
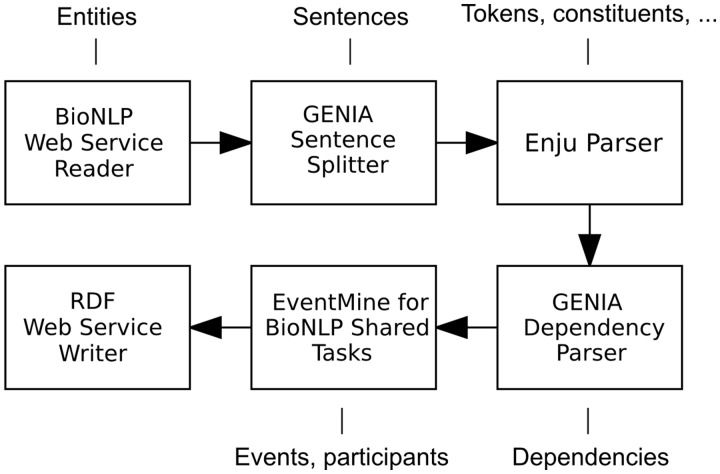
A Web service-enabled workflow built in Argo for biological event extraction. The workflow accepts REST calls with data in BioNLP format and produces RDF output. The callouts show component-specific output annotation types that are relevant for this workflow.

To showcase a heterogeneous combination of input and output formats, the workflow described below and illustrated in Figure 2 begins with a BioNLP Web Service Reader and terminates with a RDF Web Service Writer. Each document in the BioNLP shared task format is initially segmented into sentences by the GENIA Sentence Splitter. Each sentence is then processed by the Enju Parser (27) and GENIA Dependency Parser (28), which provide the next component, EventMine, with deep syntactic analyses. EventMine is a machine learning-based event extraction system that performs a series of classifications for event trigger recognition, participant identification and role assignment. Additionally, it is capable of resolving coreferences (29) and recognizing event modifications such as negation and speculation (30). It achieved the best performance on the BioNLP Shared Task GENIA 2011 (GE’11), Epigenetics and Post-translational Modifications (EPI), Infectious Diseases (ID) and Pathway Curation (PC) data sets and the second best on the Cancer Genetics (CG) data set (31). The EventMine component in Argo allows the user to choose a model tailored for a specific extraction task (one of GE, EPI, ID, PC, CG), which ultimately defines the output event types.

## Biological literature corpora in interoperable formats

For the benefit of the community, we used Argo’s format conversion capabilities and transcribed several publicly available corpora into the different formats supported by the workbench. The resulting work was made available on the Argo website (http://argo.nactem.ac.uk/bioc).

### Metabolites corpus

NaCTeM’s Metabolites corpus consists of 296 MEDLINE abstracts enriched with entity annotations corresponding to metabolites and enzymes (32). Previously used in a pilot study on yeast metabolic network reconstruction (33), the documents were manually annotated by two domain experts who marked up names of enzymes and metabolites appearing in the context of metabolic pathways. The corpus was first converted from its original MEDLINE XML format to the BioNLP format outside of Argo. We then used the SPARQL annotation editor component to transcribe it to the BioC format.

### BioNLP shared task corpora

The biennial BioNLP shared task series have produced a wide range of event-annotated corpora to support the development and evaluation of event extraction methodologies. The transcribed corpora include data sets from the ID and EPI tasks ran in the 2011 edition of the series, the CG and PC tasks ran in the 2013 edition, as well as the GENIA tasks (GE’11 and GE’13) ran in both editions.

The conversion from the original BioNLP format to BioC is more complex than that from the Metabolites corpus and goes beyond simple span-of-text annotations. The BioC format proved to be sufficiently versatile to fully and unambiguously transcribe the BioNLP corpora. Table 2 shows the example snippets of BioC syntax for each of the BioNLP annotations. Entities and event trigger words are transcribed to BioC annotations, whereas equivalent entities and events are transcribed to BioC relations. To disambiguate between them, we added infon elements with the key ‘type’ and values appropriate to what the encompassing elements represent. Infons are also used to encode event modifications (negation, speculation). Multiple coreference annotations (specific to the GENIA corpora) are combined into a single BioC relation if they share the same subject.

**Table 2.** Examples of the transcription of BioNLP annotations into BioC XML format

Annotation category	BioNLP annotation
	BioC transcription
Entities	T1 Protein 19 49 interferon regulatory factor 4
	
	<annotation id="T1"><infon key="typeUri">uima:ts:uk.ac . . . . bionlpst.Entity</infon><infon key="type">Entity</infon><infon key="category">Protein</infon><location offset="19” length="30"/><text>interferon regulatory factor 4</text></annotation>

Events with modifications	T11 Gene_expression 55 65 expressionE2 Gene_expression:T11 Theme:T1M1 Speculation E2
	
	<annotation id="TRIGGER_55_65"><infon key="type">Trigger</infon><location offset="55” length="10"/><text>expression</text></annotation><relation id="E2"><infon key="typeUri">uima:ts:uk.ac . . . .bionlpst.Event</infon><infon key="type">Event</infon><infon key="category">Gene_expression</infon><infon key="negation">false</infon><infon key="speculation">true</infon><node refid="TRIGGER_55_65” role="EventTrigger"/><node refid="T1” role="Theme"/></relation>

Equivalent entities	* Equiv T2 T3
	
	<relation id="EE53"><infon key="typeUri">uima:ts:uk . . . .EquivalentEntities</infon><infon key="type">Equivalent</infon><node refid="T2” role=""/><node refid="T3” role=""/></relation>

Coreferences (GENIA corpora)	R1 Coreference Subject:T13 Object:T3R2 Coreference Subject:T13 Object:T4R3 Coreference Subject:T13 Object:T5
	
	<relation id="RT13"><infon key="typeUri">uima:ts:uk.ac . . . .cas.bionlpst.Relation</infon><infon key="type">Coreference</infon><node refid="T13” role="Subject"/><node refid="T3” role="Object"/><node refid="T4” role="Object"/><node refid="T5” role="Object"/></relation>

Similar conversions of the BioNLP Shared Tasks corpora to BioC were demonstrated by others (25, 34). In comparison, we increased the semantic interoperability of (mainly syntactically interoperable) BioC format by allocating type URIs for each annotation. The URIs are built from qualified names of annotation types of Argo’s type systems. For instance, the UIMA built-in type uima. tcas.Annotation has the URI uima:ts: uima.tcas.Annotation (BioNLP-specific examples are shown in Table 2). The ‘uima:’ prefix is the URIs’ scheme name added to make the type names comply with the URI specification and to hint the source of type definitions. The ‘ts’ part of the URIs stands for ‘type system’ and is added to emphasize and disambiguate that the rest of a URI is related to the type system aspect of UIMA (as opposed to, e.g. data structures or analytics).

The introduction of URIs augments the semantics of annotations in BioC for humans and partially for machines. In the latter case, the URIs are of use to only those computing routines that are aware of this addition, as type URIs are not part of the BioC specification.

## Analytics evaluation

We evaluated the efficiency and effectiveness of our methods by participating in a shared task, referred to as the CTD track, of the BioCreative IV challenge. The track was organized specifically to encourage members of the text-mining community to develop interoperable automatic tools that can possibly assist in the curation of the CTD (35). This database is a publicly available resource that integrates information on chemicals, genes and diseases curated from scientific literature, aiming to foster understanding of the means by which drugs and chemicals affect human health. Relationships between entities (e.g. chemical-gene, chemical-disease and gene-disease) are stored in the database by means of manual curation. The CTD track required the preparation of RESTful Web services capable of accepting input documents in the BioC format, and returning, within a minimal amount of time, enriched versions containing annotations for unique concepts of one of four types, namely, chemicals, genes, diseases and action terms. We addressed this challenge by using algorithms for sequence labelling (for identifying chemicals, genes and diseases) and multiclass, multilabel classification (for identifying action terms), while leveraging relevant resources such as the CTD vocabularies and other ontologies/databases (We note that during our participation in the challenge Argo did not yet feature Web service-enabled components, and the CTD Web services were created outside of the workbench).

The organizers provided a development corpus of 1112 PubMed abstracts encoded in the BioC XML format. Each abstract consisted of a list of unique chemicals, genes, diseases and action terms that were manually identified by domain experts. The annotations did not include specific textual locations of the concepts. Furthermore, they corresponded to the preferred names of the concepts in the CTD vocabularies, rather than the surface forms appearing in actual text.

The automatic annotation methods, described later in the text, heavily relied on several external dictionaries. Apart from the chemical, gene and disease vocabularies available in CTD, we also used databases listed in Table 3.

**Table 3.** External databases used as dictionaries by the proposed NERs

Concept type	External databases
Chemical	Chemical Entities of Biological Interest (ChEBI) (36), DrugBank (37), Joint Chemical Dictionary (38), PubChem Compound (39)
Gene	UniProt (40), NCBI EntrezGene (41), GeneLexicon (42), Human Genome Organisation Ontology (HUGO) (43)
Disease	Medical Subject Headings (MeSH) (44), Unified Medical Language System (UMLS) (45), Disease Ontology (46), Online Mendelian Inheritance in Man (OMIM) Ontology (47)
Action term	BioLexicon (48)

### Chemical, gene and disease recognizers

We cast the problem of recognizing chemicals, genes and diseases as a named entity recognition (NER) task. Specifically, we modelled the data using conditional random fields (CRFs) (49).

As the development corpus did not contain locations of entities nor the exact forms in which they appear in the documents, the first challenge we addressed was the generation of silver-annotated corpora suitable for the named entity recognition task. Leveraging the CTD vocabularies, we determined the locations of chemical, gene and disease mentions in the abstracts using case-insensitive exact string matching. This, however, introduced a considerable amount of noise because of the ambiguity of certain names (e.g. the chemical ‘lead’ matches verbs of the same form). To mitigate this problem, we exploited the testing facility (http://bc.ctdbase.org/ws) provided by the CTD track organizers to identify and filter out false-positive results returned for each document. The remaining entities (i.e. the true-positive results) were then used in silver-annotating the documents in the corpus with their specific locations in text.

We observed, however, that in silver-annotating the corpus for diseases, many of the names in the gold standard annotations were missed because of the various ways in which they are expressed in text. For instance, the name ‘leukopenia’ appears as a curated disease for one of the abstracts and while the adjective ‘leukopenic’ appears in text, the name itself (or any of its synonyms) does not. To capture such cases, we developed a heuristic, approximate string matching method based on overlapping stemmed tokens. This algorithm is based on the steps outlined in Table 4 and is applied to both the dictionary entries in the CTD disease vocabulary as well as the noun phrases in text. For each noun phrase-dictionary entry pair, a score is computed based on the number of common tokens. If the score is greater than an established threshold, the matching tokens are silver-annotated in text.

**Table 4.** Approximate string matching algorithm applied to produce silver annotations

Step	Phrase in text	CTD entry
Input	injured by stun gun	Stun Gun Injury
Case normalization	injured by stun gun	stun gun injury
Stop word removal	injured stun gun	stun gun injury
Stemming	injur stun gun	stun gun injur
Reordering	gun injur stun	gun injur stun

As an initial step to the training of CRF models, the abstracts were pre-processed by sentence splitting [using the MEDLINE sentence model in LingPipe (http://alias-i.com/lingpipe)], tokenization [using OSCAR4 (50)] and part-of-speech and chunk tagging [using GENIA Tagger (51)]. The NERsuite package (http://nersuite.nlplab.org), our CRF implementation of choice, generates lexical, orthographic, syntactic and dictionary match features that were used in the training of the CRF models. In tagging named entities in input abstracts, NERsuite generates the same set of features and assigns begin-inside-outside labels to the token sequences using the trained models. These labels are then processed to produce responses, i.e. text spans corresponding to recognized concepts.

Two items relevant to how the tools were evaluated came to our attention during the development phase of the shared task: (i) although normalization of entities to the CTD vocabularies was not a requirement, the official testing facility calculated the number of successful matches by attempting to map the responses to the CTD preferred names in the gold standard annotations directly or indirectly through synonyms, using case-insensitive exact string matching; (ii) the task organizers communicated to the participants that while a balance between precision and recall is desirable, optimal recall was preferable as far as actual CTD curation was concerned.

Considering these points, we incorporated a check for responses that could not be mapped to CTD preferred names. In such cases, we applied the previously mentioned heuristic method on both the response annotation and CTD entries to retrieve and return the most similar CTD name or synonym (i.e. the highest scoring entry).

### Action term recognizer

Unlike chemicals, genes and diseases, CTD action terms were expressed in text much less explicitly. Action terms such as ‘response to substance’ would rarely appear verbatim in actual text, with authors expressing the same idea by instead saying that ‘A affects B in some manner C’. For this reason, and considering that there was a relatively small set of possible CTD action terms, we decided to cast the problem as a multiclass, multilabel classification task, in which each abstract could be labelled with any number of action terms (from a set of 53) depending on the types of chemical-gene interactions that particular abstract pertains to.

Each abstract underwent the same pre-processing pipeline as the one applied for the other categories described in the previous section. Using a one-versus-all approach, we used support vector machines to train a total of 53 different models (i.e. one for each of the 53 CTD action terms). The feature set used in the training and classification included (1) verb variant matches based on BioLexicon entries, and (2) co-occurrence (and proximity) of chemical and gene names with a biomedical verb variant. Features of the first type were represented as booleans, while those of the second type were normalized weights accumulated based on the number of co-occurrences. To facilitate the extraction of the second feature type, chemical and gene names were tagged automatically using the CRF models previously described. If the prediction returned by any of the 53 models was greater than an established threshold, the document was labelled with the CTD action term corresponding to that model.

## Results

The CTD track organizers carried out the official evaluation of the automatic tools using a test corpus consisting of 510 PubMed abstracts. Reported in Table 5 are the official (released by the organizers) results obtained by our recognizers, measured in terms of standard performance metrics (micro-averaged precision, recall and *F*-score) and average processing times.

**Table 5.** Official BioCreative IV evaluation results for NaCTeM’s CTD Web services

Category	Precision(%)	Recall(%)	F-score(%)	Average response time (sec.)
Chemical	75.24	73.41	74.31	0.77
Gene	53.61	70.86	61.04	0.80
Disease	34.67	49.42	40.75	0.78
Action term	34.53	50.72	41.09	0.92

Each of our recognizers performed well with average response times of less than a second. This is especially true for our chemical and gene recognizers that were also ranked the highest (out of 10 and 9 teams, respectively) in terms of *F*-score. The organizers also took into consideration combined micro-averages, i.e. the average of the *F*-scores from all concept categories. Our recognizers achieved the highest combined average out of 10 teams (22).

To evaluate the utility of external dictionaries, we compared the performance of our solution with several other versions that involved different number of dictionaries. Table 6 summarizes the results (Although both sets of results presented in Tables 5 and 6 were obtained using the same official online testing facility, there are minor discrepancies between the two sets, which is the consequence of a few changes in the gold standard corpora applied by the BioCreative organizers over time). The difference in *F*-score between the set-ups that do not use dictionaries and the one that makes use of all the dictionaries is statistically significant and ranges from 2.2 to 2.8% points for the three categories: chemical, gene and disease. The addition of the external vocabularies (listed in Table 3) improves the performance in all cases over using only the CTD vocabularies; however, the difference is only statistically significant for chemicals.

**Table 6.** Contribution of dictionaries to the performance of the proposed NERs

Dictionaries	Chemical			Gene			Disease		
	Precision	Recall	*F*-score	Precision	Recall	*F*-score	Precision	Recall	*F*-score
None	76.38	67.28	**71.54	53.77	64.23	**58.54	33.87	44.85	**38.59
CTD only	74.59	72.40	*73.48	53.28	68.87	60.08	34.35	49.10	40.42
All	75.24	73.41	74.31	53.61	70.92	61.06	34.67	49.52	40.79

*Note:* The difference in F-score between the NERs using all dictionaries and the other setups is statistically significant for cells marked with *(0.01 < *P*-value <0.05) and ** (*P*-value <0.01).

Values in percentages.

We also compared the performance of our NERs trained on our generated silver corpus against the same NERs trained on other, domain-related gold standard corpora. We used the CHEMDNER corpus (52) prepared for another track of BioCreative IV, the Gene Mention corpus (53) prepared for BioCreative II and the NCBI Disease corpus (54). The results, summarized in Table 7, show that using our silver-annotation technique is superior to training NERs on established gold standard corpora in terms of precision and *F*-score. This demonstrates that despite training on similar-domain data, the CTD corpus is much more specialized. This is especially true for chemicals and genes. For instance, although the recall for chemicals is better when trained on the CHEMDNER corpus (by ∼10% points), the corpus includes a far greater range of chemical types, which has much more negative impact on precision (that drops by ∼34% points).

**Table 7.** Performance gain of the proposed NERs (with all dictionaries) trained on the created silver corpus against the same NERs trained on domain-related, gold standard corpora

Category	Gold standard corpus	Precision	Recall	F-score
Chemical	BioCreative IV CHEMDNER	+34.11	−10.40	+19.13
Gene	BioCreative II Gene Mention	+23.91	−4.28	+18.48
Disease	NCBI Disease	+3.30	+0.74	+2.60

*Note: F*-score gain is statistically significant (*P*-value <0.01) for all categories. Values in percentage points.

## Conclusions

Web-based Argo is a one-stop workbench with a convenient graphical user interface for creating text mining Web services for the processing of biological literature. The main advantage of the workbench over other platforms is its unique mechanism for customizing Web services that involves the formulation of user-defined processing tasks and a selection of the services’ input and output formats. The discussed formats, BioC, BioNLP, XMI and RDF, represent both domain-specific and generic representations and include well established as well as emerging specifications. The formats also differ in their support for syntactic and semantic interoperability. Because of its specific and limited, applicability, BioNLP defines precise syntax and semantics. BioC defines mostly syntactic interoperability with some rudimentary semantic definitions. We have proposed to increase the semantic interoperability of this format by including URIs of annotation types. This, however, can only be fully accomplished if the URI requirement becomes part of the format’s syntax. Most expressive, yet fully interoperable are the generic XMI and RDF formats. Their semantics are ensured by well-defined type systems underpinning any UIMA-based platform, including Argo.

The customizable input and output formats are complemented by highly effective and efficient biology-relevant analytics available in Argo. The superiority of the analytics was validated at an international challenge where our solutions generally outperformed those submitted by other groups, based on combined average scores.

## Funding

This work was partially funded by Europe PubMed Central funders led by Wellcome Trust, UK. RB was financially supported by the University of the Philippines and the Engineering Research and Development for Technology faculty development program. Funding for open access charge: The University of Manchester's Wellcome Trust award.
